# Involvement of baroreflex deficiency in the age-related loss of estrogen efficacy against cerebral ischemia

**DOI:** 10.3389/fnagi.2023.1167170

**Published:** 2023-05-02

**Authors:** Lei Wang, Jia Wang, Qing Shan, He Shu, Jin-Min Guo

**Affiliations:** ^1^Department of Orthopedics, 960th Hospital of PLA, Jinan, Shandong, China; ^2^Health Service Department, 960th Hospital of PLA, Jinan, Shandong, China; ^3^Department of Clinical Pharmacy, Weifang Medical University, Weifang, Shandong, China; ^4^Department of Clinical Pharmacy, 960th Hospital of PLA, Jinan, Shandong, China

**Keywords:** arterial baroreflex, estrogens, ischemic stroke, estrogen replacement therapy, α7 nicotinic acetylcholine receptor

## Abstract

For post-menopausal women, stroke is complicated by the variable effects of estrogen therapy and the age-related therapeutic consequences involved. Estrogen therapy has been shown to have an age-dimorphic effect, which is neuroprotective in young females, but non-neuroprotective, even neurotoxic in acyclic females. We hypothesized that arterial baroreflex (ABR) and its downstream acetylcholine-α7 nicotinic acetylcholine receptor (α7nAChR) anti-inflammatory pathways are involved in estrogen efficacy toward cerebral ischemic damage. Our data showed that estrogen supplements contributed to ABR improvement and neuroprotection in adult, not aged, ovariectomized (OVX) rats. In adult rats, OVX-induced estrogen deficiency aggravated middle cerebral artery occlusion (MCAO), which induced brain infarction and reduced ABR function, with decreased α7nAChR expression of the brain and exaggerated inflammation following MCAO; these effects were significantly prevented by supplementation with estrogen. ABR impairment by sinoaortic denervation partly attenuated the estrogen effect on baroreflex sensitivity (BRS) and ischemic damage in adult rats, as well as α7nAChR expression and inflammatory response. These data suggested that ABR and acetylcholine-α7nAChR anti-inflammatory pathways are involved in the neuroprotection of estrogen in adult OVX rats. In contrast, aged rats exhibited more severe ischemic damage and inflammatory response than adult rats, as well as poorer baroreflex function and lower α7nAChR expression. Estrogen supplements did not improve BRS or confer neuroprotection in aged rats without affecting brain α7nAChR and post-ischemic inflammation. Most importantly, ketanserin restored ABR function and significantly postponed the onset of stroke in aged female strokeprone spontaneously hypertensive rats, whereas estrogen treatment failed to delay the development of stroke. Our findings reveal that estrogen is protective against ischemic stroke (IS) in adult female rats and that ABR played a role in this beneficial action. Dysfunction of ABR and unresponsiveness to estrogen in aged female rats may contribute to a reduced estrogen efficacy against cerebral ischemia.

## Introduction

Stroke causes disability and even death to a great extent globally (Wan et al., 2019; Li et al., 2021; Xu et al., 2022b), and the incidence of stroke increases with advancing age, especially ischemic stroke (IS), which accounts for approximately 80% of cases; thus, it is crucial to prevent its occurrence (Wan et al., 2019; Li et al., 2021). A complete understanding of stroke-related risk factors, including hypertension, hyperlipidemia, and baroreflex dysfunction, is essential for the target population.

Gender plays a prominent role in predicting the risk of stroke. Epidemiology studies have shown that elderly women can experience more severe strokes, poor recovery, and increased long-term disability than their male counterparts, whereas pre-menopausal women suffer less frequently from stroke than age-matched men (Bell et al., 2013; Manson et al., 2013; Canonico et al., 2016; Weill et al., 2016; Cespedes Rubio et al., 2018; Branyan and Sohrabji, 2020; Jett et al., 2022; Tang et al., 2022). Generally, estrogen is responsible for protection against relative stroke reported in younger and pre-menopausal women; however, unexpectedly, older women subjected to estrogen therapy showed an increase in susceptibility to stroke (Manson et al., 2013; Engler-Chiurazzi et al., 2017; Cespedes Rubio et al., 2018). This age-related dimorphic effect of estrogen was also demonstrated in experimental studies, revealing an estrogen neuroprotective effect in young ovariectomized rats but a non-neuroprotective, even neurotoxic, effect in acyclic female rats (Selvamani and Sohrabji, 2010; Leon et al., 2012; Selvaraj et al., 2018; Ma et al., 2020; Park et al., 2020; Oppong-Gyebi et al., 2022). Thus, it has been proposed that the estrogen-therapy effect needs to take age-related changes into account, which cannot be considered in isolation. A gradual loss in estrogen effectiveness may be observed because of the age-dependent loss of key downstream agents that define the transition of estrogen between the neuroprotective and neurotoxic effects (Sohrabji et al., 2013b; Xu et al., 2022a). However, relevant studies are scarce, and the underlying mechanism remains unclear. Therefore, it becomes even more important to fully understand the functions of estrogen in the elderly and to explore the mechanisms behind these apparently diverging impacts deeply.

Arterial baroreflex (ABR) is a significant predictor of the development of stroke and its prognosis, apart from being an essential prevention strategy for stroke (Liu et al., 2007). Impaired baroreflex sensitivity (BRS) is prevalent in acute stroke and indicates poor prognosis, whereas restoring the BRS is protective against stroke (Liu et al., 2007, 2013). The acetylcholine-α7 nicotinic acetylcholine receptor (α7nAChR) anti-inflammatory pathway was reported as ABR downstream and is involved in its stroke-protective action (Li et al., 2021). As one ABR arm, vagal nerve activation has the ability to suppress inflammation, mainly through acetylcholine acting at the acetylcholine-α7nAChR. Previous studies reported that ABR exhibited a natural decline with age (Irigoyen and Krieger, 1998). Moreover, accumulating evidence indicates the critical role of estrogen in modulating the autonomic tone and BRS (Saleh and Connell, 2000; Pourshanazari et al., 2014). Thus, we hypothesize that ABR may be involved in the neuroprotection of estrogen, and an age-dependent dysfunction of ABR may contribute to the gradual loss of estrogen effectiveness as a neuroprotectant.

In this study, young and aged rats were used to examine the efficacy of estrogen on ischemic damage as well as on stroke development. Subsequently, a series of experiments were performed to examine ABR potential and its downstream pathway, namely, the acetylcholine-α7nAChR anti-inflammatory pathway.

## Materials and methods

### Animals

The animal center of Second Military Medical University provided female 36-week-old strokeprone spontaneously hypertensive rats (SHRSP), female 12-week-old (adult), and 70-week-old (aged) SD rats, which were subjected to a temperature of 23–25°C with a 12:12 light:dark (L:D) cycle with lights turned on from 8:00 AM to 8:00 PM, apart from accessing standard food and water freely.

### Reagents and drugs

In all, 0.3 mg/kg/d of ketanserin (Ket, Janssen Pharmaceutica, Beerse, Belgium) was used for 3 weeks as a positive control (intragastric) for improving ABR (Yu et al., 2013). In addition, 17β-Estradiol was procured from Sigma-Aldrich (St. Louis, MO, United States). Anti-α7nAChR and anti-GAPDH antibodies were procured from Cell Signaling Technology Inc. (Danvers, MA, United States).

### Ovariectomy and estrogen replacement

By dividing the adult rats into the sham (with the vehicle), OVX (with the vehicle), and OVX + E (with 17β-estradiol) groups, both estrogen deficiency and replacement conditions were established. Ovariectomy (OVX) was performed by a dorsolateral incision according to the description given by Leon et al. (2012). In brief, the animals were anesthetized first and then two small lateral abdominal incisions were made to expose the right and left uterus horns, succeeded by the careful removal of the ovaries, leaving the uterus intact. As regards the sham group, the same operation was performed, but the ovaries remained intact. After a 5-day OVX, animals were injected daily for 28 days with estrogen subcutaneously (500 μg/kg in rats and mice, diluted in sesame oil solution) or vehicle at the back of the neck (Cai et al., 2014).

For the confirmation of estrogen deficiency and the effectiveness of replacement, serum estrogen levels were assessed. In brief, using an overdose of pentobarbital sodium, the animals were anesthetized, collecting blood from the ophthalmic artery. ELISA kit (Xi-Tang BioTech, Shanghai, China) was utilized for measuring the estrogen, progestin, and testosterone serum levels.

### Blood pressure and BRS measurement

In conscious, freely moving rats, systolic blood pressure (SBP), diastolic blood pressure (DBP), and heart period were constantly monitored, according to Liu et al. (2013). After 3-day training, at least three evaluations were performed on each rat every session, expressing ABR function as a BRS value, which was determined according to Liu et al. (2013). In addition, at sacrifice and after removing the uterus, each rat was weighed.

### Middle cerebral artery occlusion

Based on Guo et al. (2013), the MCAO surgery took place in rats, and 2 h after the operation, the occluding filament was withdrawn to allow reperfusion. The rats were sacrificed 1 day after MCAO to perform several examinations after neurological deficit scoring, which was performed using a 5-point scale (Guo et al., 2013). A brain-cutting matrix (ASI Instruments, Warren, MI, United States) was utilized to prepare brain slices, and 2,3,5-triphenyltetrazolium chloride (TTC) staining was utilized to determine the infarct size, which was expressed as the contralateral hemisphere percentage.

### Behavioral assessment and morphological examination

The female SHRSP had been administered a mix of estrogen (500 μg/kg/d), Ket (0.3 mg/kg/d), and vehicle consecutively with food and the stroke survival time and behavioral signs were monitored according to the instructions of Liu et al. (2013). To discard the non-stroke dead, the extracted brain upon death was examined for hemorrhage, edema, and infarction.

### Sinoaortic denervation

According to Liu et al. (2007), SAD was performed as follows: in brief, the rats were subjected to a mix of intraperitoneal ketamine and diazepam (50 mg/kg + 5 mg/kg), plus intraperitoneal atropine sulfate (0.5 mg/kg) and intramuscular procaine benzylpenicillin (60,000 U). The superior laryngeal nerves were transected near the vagus nerves on both sides, and the superior cervical ganglia and a small sympathetic trunk section were removed. The aortic depressor nerves were also transected on both sides. The carotid sinus baroreceptors were mechanically stripped at the carotid bifurcation and its branches. In total, 10% of phenol (in 95% ethanol) was used for treating the external, internal, and common carotid arteries alongside the occipital artery. Meanwhile, a midline neck incision was made and bilateral neck muscle isolation took place in the sham operation. SAD was conducted 1 month prior to OVX treatment.

### Proinflammatory cytokine measurement

An enzyme-linked immunosorbent assay was used by an automatic microplate reader (Infinite M200; Tecan Austria GmbH, Austria) for determining the tumor necrosis factor α (TNF-α) and interleukin-1β (IL-1β) serum levels and collected 12 h after MCAO.

### Western blot analysis

The expression of α7nAChR was evaluated after a 4-week estrogen pre-treatment. According to Guo et al. (2013), brain tissue proteins were extracted. After subjecting equivalent protein amounts (50 μg) to SDS-PAGE, they were then transferred to a polyvinylidene fluoride membrane. After being blocked for 2 h with 5% BSA at room temperature, the membranes were incubated with an anti-α7-nAChR antibody (1:1000) or an anti-GAPDH antibody (1:20,000) before incubation with IRDye 800CW-conjugated secondary antibody (Rockland Immunochemicals, Inc.). The Odyssey Infrared Imaging System (Li-Cor Biosciences, Lincoln, NE, United States) was utilized for capturing the images, and ImageJ software (NIH) was used for analyzing them.

### Statistical analysis

The one-way analysis of variance (ANOVA) was performed to analyze the data, which were reported as mean ± SD. By performing the Kaplan–Meier analysis, survival probabilities were estimated, while the survival curve equality was evaluated by the log-rank test. A value of *p* < 0.05 indicated statistical significance.

## Results

### Estrogen supplements significantly increased serum estrogen levels and attenuated the uterine weight gain induced in OVX adult and aged rats

**Figure 1:**
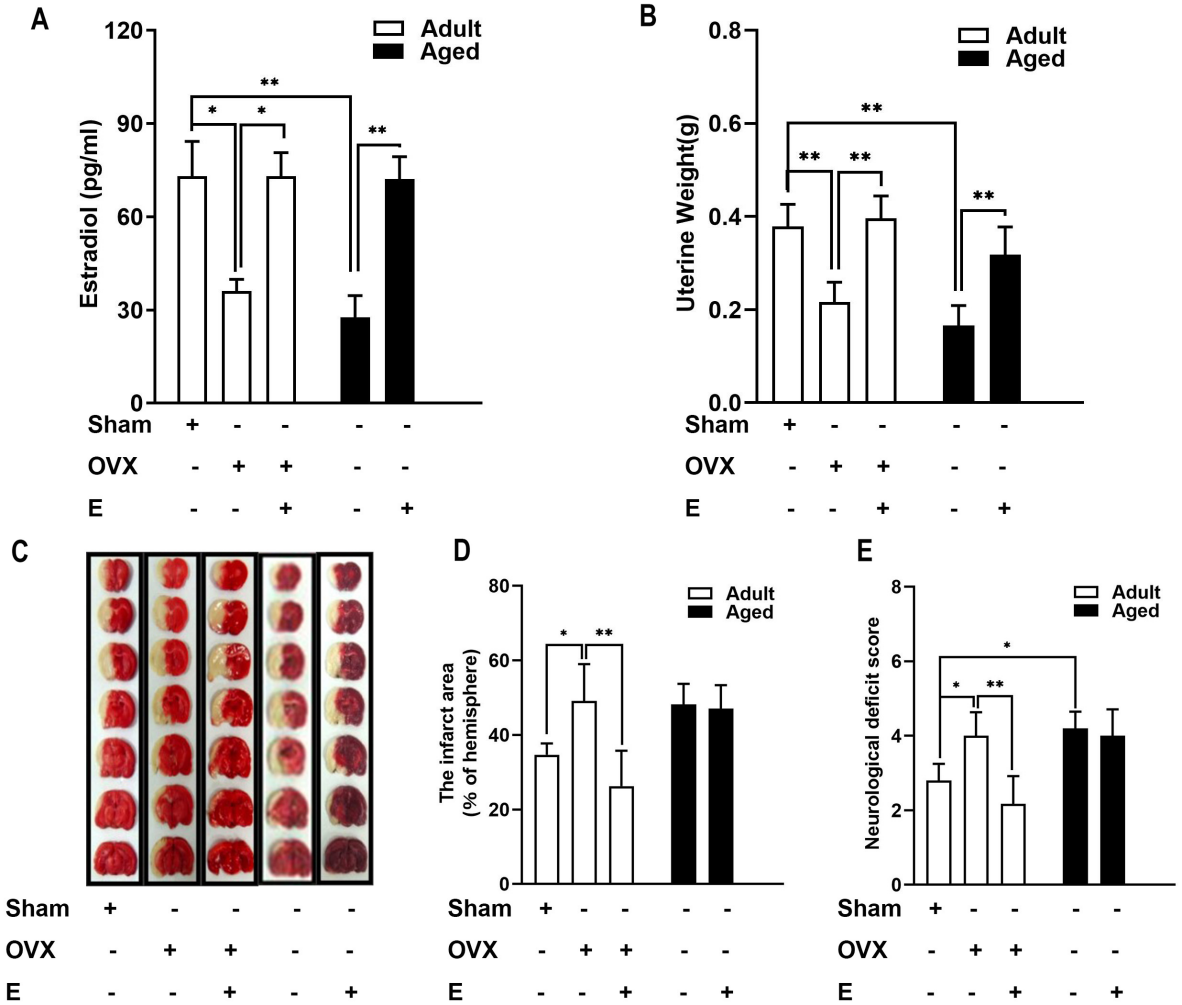
Estrogen conferred neuroprotection in adult OVX rats but not in aged rats. **(A)** In adult rats, OVX led to a decrease in serum estrogen levels, whereas estrogen supplements (500 μg/kg, daily subcutaneous injections) for 28 days recovered the hormonal level. In aged rats, the serum estrogen level is very low, and the administration of estrogen reversed the hypo-estrogen condition. **(B)** Elevated uterine weight was exhibited after the administration of estrogen in adult OVX and aged rats. **(C–E)** In adult rats, estrogen deficiency induced by OVX led to a substantial enlargement in MCAO-induced infarction volume and neurological deficits compared with the sham group. This effect of OVX was significantly prevented by supplementation with estrogen. Ischemic damage was more severe than in aged rats than in adult rats, and estrogen supplements did not confer neuroprotection. C shows representative rostrocaudal series of TTC-stained coronal sections 1 day after MCAO. Data were analyzed by the one-way analysis of variance (ANOVA) followed by least significant difference *t*-test, *n* = 5–6 in each group. *p < 0.05, **p < 0.01.

To validate the efficacy of estrogen supplements in SD rats, serum estrogen levels were used as measurements, which were more significantly reduced in adult OVX rats, suggesting estrogen deficiency. Serum estrogen level was 1.91 times higher in the OVX + E group compared to the OVX group, indicating the successful treatment of estrogen replacement (Figure 1A, *p* < 0.05). In aged rats, serum estrogen levels were low, consistent with the physiological status of hypoestrogenemia after menopause. Exogenous administration of estrogen reversed hypoestrogenism in aged rats (Figure 1A, *p* < 0.01). In addition, consistent with its known effects, treatment with estrogen restored the OVX-induced decrease in uterine weight in adult rats (Figure 1B, *p <* 0.001). In aged rats, similar results were observed; an elevated weight was exhibited after administration of estrogen (Figure 1B, *p <* 0.001). These results suggested that exogenous administration of estrogen improved estrogen deficiency both in OVX adults and in aged rats.

### Estrogen supplements conferred neuroprotection in adult OVX rats but not in aged rats

According to the experiments conducted using MCAO rats to determine the effect of estrogen supplements on IS, in adult rats, an OVX-induced estrogen deficiency caused the substantial enlargement of MCAO-induced infarction volume (by 41.73%) and neurological deficits more than the sham group (Figures 1C–E, *p <* 0.05). This OVX influence was prevented significantly by supplementation with estrogen (Figures 1C–E). Ischemic damage was more severe in aged rats than in adult rats, and estrogen supplements did not confer neuroprotection (Figures 1C–E). Consistent with previous studies (Selvamani and Sohrabji, 2010; Ma et al., 2020), estrogen’s age-dimorphic effect on stroke was observed, and neuroprotection of estrogen was exhibited only in adult OVX rats but not in aged rats.

### Estrogen supplement improved ABR function in adult OVX rats but not in aged rats

**Figure 2:**
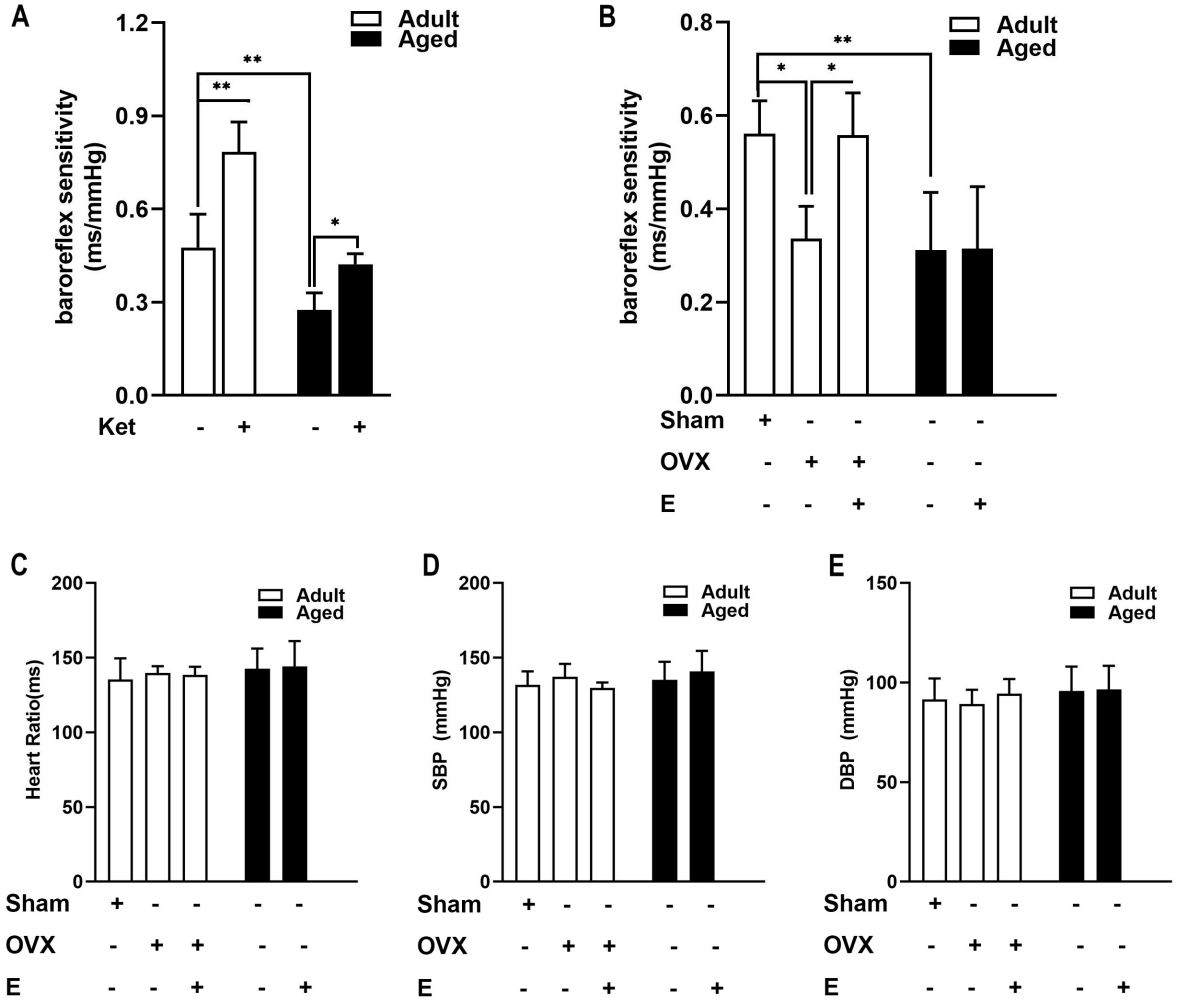
Estrogen supplements improved arterial baroreflex (ABR) function in adult OVX rats but not in aged rats. **(A)** Ketansarin (Ket) (0.3 mg/kg/d), as a positive control group, significantly increased BRS in both adult and aged rats. **(B)** In adult rats, OVX led to a baroreflex dysfunction and exhibited a reduced BRS value, whereas estrogen replacement restored BRS values. The function of arterial baroreflex was weaker in aged rats than in adult rats, and estrogen supplements failed to improve BRS. **(C–E)** Estrogen pretreatment did not change systolic blood pressure (SBP), diastolic blood pressure (DBP) and heart period in adult and aged rats. Data were analyzed by the one-way analysis of variance (ANOVA) followed by least significant difference *t*-test, *n* = 5–6 in each group. *p < 0.05, **p < 0.01.

To determine the age-related decline in key agents that complement or collaborate with estrogen, the known stroke risk factors (BRS and blood pressure) were evaluated in conscious rats after a 4-week estrogen pre-treatment. As shown in Figure 2A, 0.3 mg/kg/d of Ket, as a positive control group, led to a significant increase in BRS in both adult (by 66.35%, *p <* 0.001) and aged rats (by 48.94%, *p* < 0.05), with no effect on blood pressure and heart period (data not shown) more than the control. As regards ABR function, estrogen supplements exhibited a similar effect with Ket in adult OVX rats, but not in aged rats. In adult rats, OVX led to a baroreflex dysfunction, exhibiting reduced BRS, which was then restored by estrogen replacement (Figure 2B, *p* < 0.05). On the contrary, in aged rats, ABR function was poorer than that in adult rats, indicating an age-related effect (Figure 2B, *p* < 0.01). More importantly, estrogen supplements did not improve BRS in aged rats (Figure 2B). These results indicated that a decline in arterial BRS and a loss of estrogen efficacy to improve ABR function were exhibited in aged rats. Apart from this, we also determined the blood pressure. These results indicated that estrogen-regulated α7nAChR expression in an ABR dependent manner (Figures 2C–E).

### Treatment with estrogen did not delay the onset of stroke in aged SHRSP

**Figure 3:**
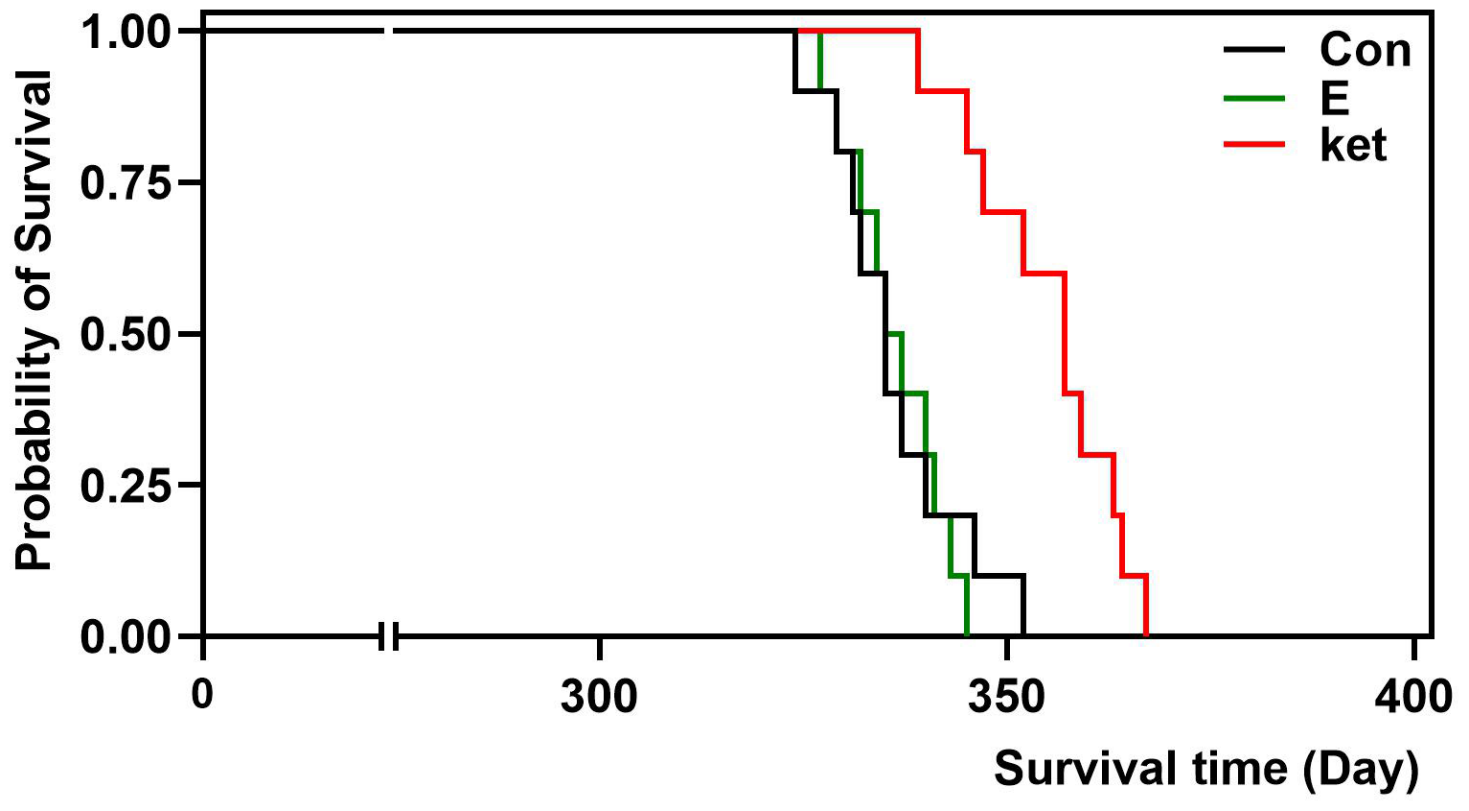
Treatment with estrogen did not delay the onset of stroke in aged rats. Lifelong treatment of Ket (0.3 mg/kg/d) postponed the stroke death of SHRSP (log-rank testing *χ*^2^ = 13.49, *p* = 0.002), whereas estrogen treatment did not postpone the stroke development of old SHRSP. *N* = 10 per group. The Kaplan–Meier analysis was used to estimate survival probabilities in the groups.

Thirty-year-old female SHRSP were subjected to estrogen (500 μg/kg/d), Ket (0.3 mg/kg/d), or vehicle treatment with animal chow. Figure 3 shows the survival time depicted with Kaplan–Meier survival curves. Ket led to a significant delay in the development of lethal stroke (log-rank *χ*^2^ = 13.49, *p* = 0.002), consistent with a previous study which showed that BRS restoration by Ket postponed the development of stroke in SHR-SPs (Liu et al., 2013). On the contrary, estrogen therapy did not postpone the development of stroke in old SHRSP, in line with the results of MCAO-induced acute ischemic damage.

### BRS correlates with the neuroprotection of estrogen from ischemic injury

**Figure 4:**
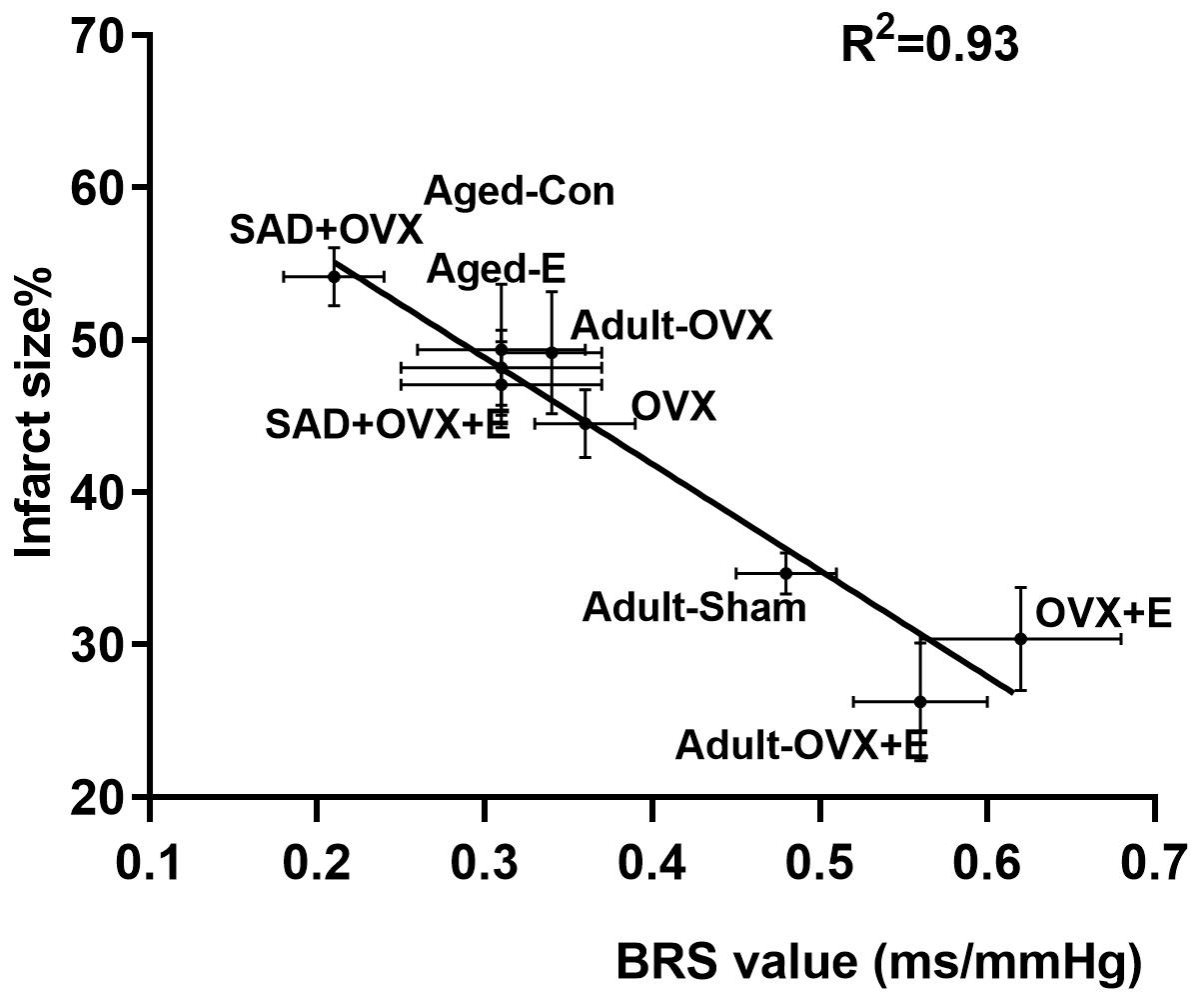
Baroflex sensitivity (BRS) value correlates with neuroprotection from ischemic injury. Measurements of BRS and ischemic cerebral damage in adult OVX and aged rats treated with vehicle or estrogen are shown is BRS as a function of the infarct size, measured by 2,3,5-TTC staining from corresponding brain samples derived from the same studies. Linear regression yielded a high inverse correlation of *R*^2^ = 0.93.

In view of the established results that estrogen induced an age-dimorphic effect in ABR and neuroprotection in the same direction, we thus proposed that ABR is involved in estrogen action. According to the above studies, BRS and cerebral ischemic damage were measured in adult OVX and aged rats treated with vehicle or estrogen. As shown in Figure 4, ABR function correlated with protection against stroke from ischemia induced by estrogen. A high inverse correlation of R^2^ = 0.93 was indicated by linear regression.

### Sad impaired ABR function and attenuated BRS improvement and estrogen neuroprotective effects in adult OVX rats

**Figure 5:**
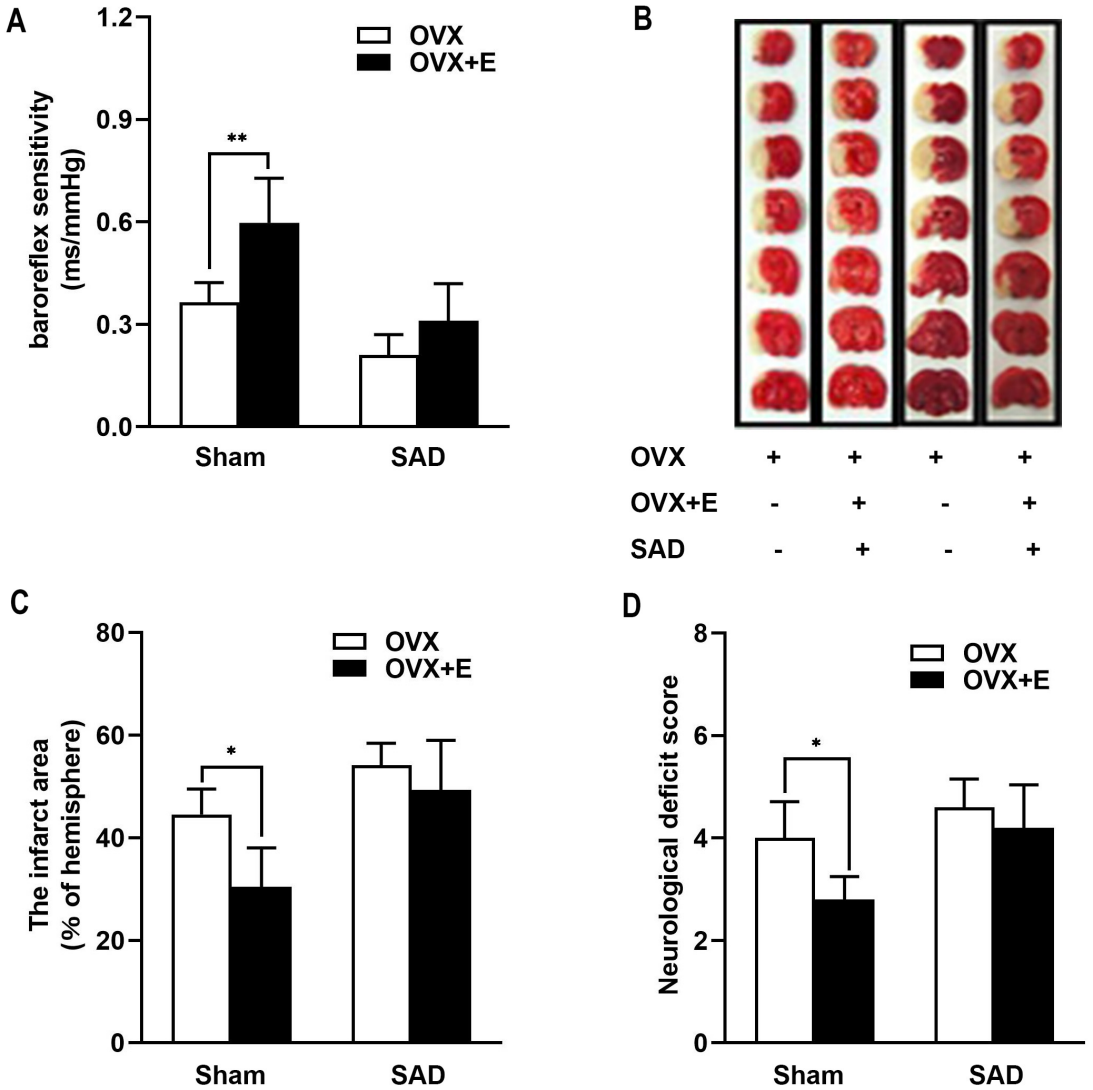
Sinoaortic denervation (SAD) impaired the function of arterial baroreflex and attenuated the improvement in BRS and neuroprotective effects of estrogen in adult OVX rats. **(A)** Estrogen supplements improved BRS compared with OVX rats, whereas SAD impaired the function of arterial baroreflex and abolished the beneficial action of estrogen on BRS. **(B,C)** Estrogen supplements reduced the infarct area in rats with an intact baroreflex by 31.72% and to a much lesser degree in SAD rats without significant difference (by 8.85%). B shows representative rostrocaudal series of TTC-stained coronal sections 1 day after MCAO. **(D)** Estrogen supplements also improved the neurological function in OVX rats with intact baroreflex but not in OVX rats with SAD. Data were analyzed by one-way analysis of variance (ANOVA) followed by least significant difference *t*-test, n = 5–6 in each group. *p < 0.05, **p < 0.01.

The hypothesis was further verified in adult OVX rats, and SAD was conducted to impair the ABR. BRS measurements were taken 1 month after SAD validated ABR loss (SAD: 0.21 ± 0.03 ms/mmHg vs. Sham group: 0.36 ± 0.03 ms/mmHg, *p* < 0.01). SAD did not affect blood pressure and heart period (data not shown). Estrogen supplements improved BRS compared with OVX rats, whereas SAD impaired ABR function and abolished the beneficial action of estrogen on BRS (Figure 5A).

Next, the role of ABR was investigated using MCAO rats. MCAO-caused infarct tended to enlarge more in SAD-subjected OVX rats than in the sham rats (54.14% ± 1.92% vs. 44.51% ± 2.24%, *p* = 0.266, Figures 5B,C). Estrogen supplements decreased the infarct area in OVX rats with intact baroreflex by 31.72%, while in SAD rats, it was reduced by a less degree to a great extent; the difference was not statistically significant (by 8.85%; Figures 5B,C). Estrogen supplements also improved the neurological function in OVX rats with intact baroreflex but not in OVX rats with SAD (Figure 5D), suggesting ABR involvement in estrogen-protective effects against IS in adult OVX rats. Then, we inferred that the loss of estrogen-improving ABR efficacy might partly account for the missed neuroprotection in aged rats.

### Estrogen supplements increased α7nAChR expression in the brain by regulating ABR in adult OVX rats

**Figure 6:**
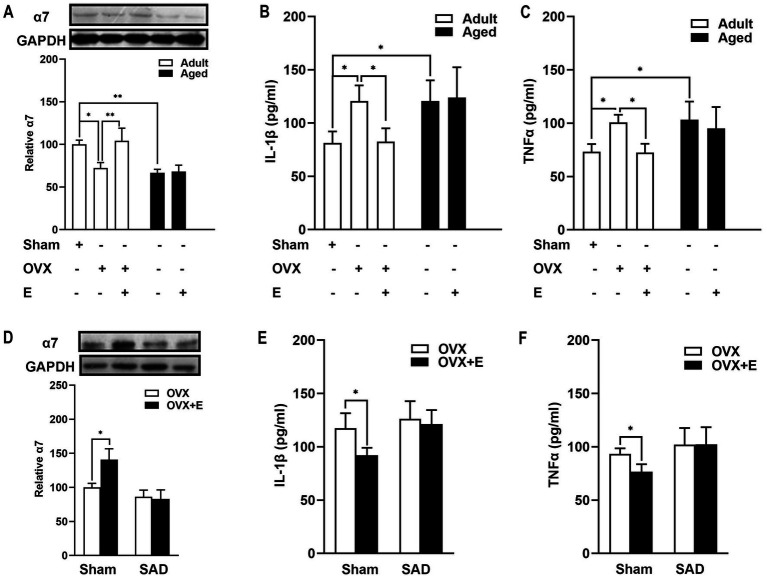
Estrogen decreased the expression of brain α7nAChR and the level of proinflammatory cytokines in an arterial baroreflex-dependent manner in adult OVX rats? **(A)** In adult rats, OVX led to a reduced expression of α7nAChR, whereas estrogen treatment reversed such a reduction.α7nAChR expression was weaker in aged rats than in adult rats and was not affected by estrogen supplements. **(B,C)** In adult rats, estrogen supplements alleviated the increase in serum TNF-α and IL-1β following MCAO in OVX rats. Whereas in aged rats, MCAO-induced inflammation was more serious than in adult rats, and estrogen treatment did not reduce the release of serum TNF-α and IL-1β. **(D)** SAD decreased the expression of α7nAChR in an OVX rat brain. Arterial baroreflex impairment by the SAD abolished the effect of the estrogen-enhancing α7nAChR. **(E,F)** SAD attenuated the reduction of proinflammatory cytokines induced by the administration of estrogen. Data were analyzed by one-way analysis of variance (ANOVA) followed by least significant difference *t*-test, n = 3–6 in each group. *p < 0.05, **p < 0.01.

Arterial baroflex (ABR) was demonstrated to play the role of neuroprotective action by promoting the neural cholinergic α7nAChR anti-inflammatory pathway (Li et al., 2021). Therefore, the expression of α7nAChR was determined. As shown in Figure 6A, estrogen deficiency, and supplementation regulated brain α7nAChR expression, and the change in α7nAChR expression acted in line with the BRS change induced by estrogen. In adult rats, OVX led to a reduced expression of α7nAChR, and estrogen treatment reversed such reduction. α7nAChR expression was weaker in aged rats than in adult rats and was not affected by an estrogen supplement (Figure 6A).

Whether ABR is involved in the estrogen-regulated α7nAChR action was further explored in adult rats. Estrogen treatment upregulated α7nAChR expression in rat brains (Figure 6D, *p* < 0.05). ABR impairment by SAD abolished the effect of the estrogen-enhancing α7nAChR (Figure 6D). SAD tended to decrease the expression of α7nAChR in OVX rats (Figure 6D). These results indicated that estrogen regulated α7nAChR expression in an ABR-involved manner.

### Estrogen supplements decreased proinflammatory cytokine levels in the serum by regulating ABR in adult OVX rats

Inflammatory cytokine profiles after MCAO were also determined. In adult rats, estrogen supplements alleviated the increase in serum TNF-α and IL-1β of OVX rats (Figures 6B,C, *p* < 0.05). MCAO-induced inflammation was more serious in aged rats than in adult rats, as evidenced by TNF-α and IL-1β serum levels (Figures 6B,C, *p* < 0.05). In aged rats, MCAO induced inflammation was more complicated than in adult rats, as evidenced by TNF-α and IL-1β serum levels (Figures 6B,C, *p* < 0.05).

Next, we further explored the role of ABR in estrogen-anti-inflammatory pathways in adult OVX rats. SAD attenuated the reduced proinflammatory cytokines induced by the administration of estrogen (Figures 6E,F), suggesting that ABR is involved in the estrogen-supplemented anti-inflammatory action after MCAO. Subsequently, the idea further inferred that estrogen anti-inflammatory action loss in aged rats is partly attributed to the failure of estrogen to regulate ABR.

## Discussion

**Figure 7:**
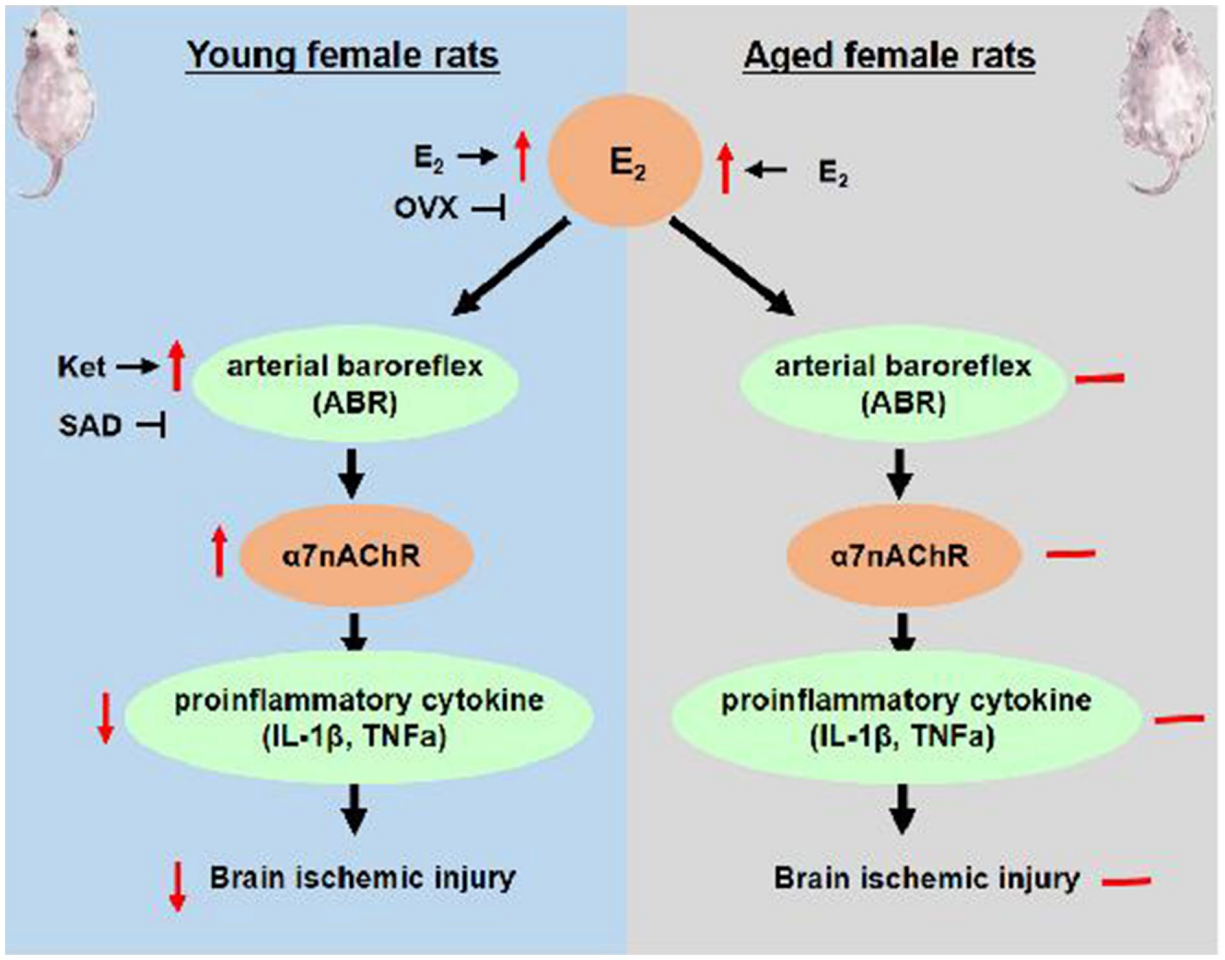
The proposed mechanism of age-dimorphic effect of estrogen on brain ischemic injury. E_2_, estrogen; OVX, ovariectomy; SAD, sinoaortic denervation.

The present data demonstrate that the age-related decline in ABR function and its responsiveness to estrogen is involved in the loss of estrogen effectiveness in the aging brain. In the present study, adult and aged female rats were used, and the estrogen age-dimorphic effect on cerebral ischemic damage was observed. In adult OVX rats, estrogen supplements improved ABR and then activated its downstream α7nAChR anti-inflammatory pathway, leading to effective prevention of ischemia-induced brain injury. However, aged rats had more severe ischemic damage and inflammatory action than adult OVX rats, as well as poorer ABR. Estrogen treatment failed to attenuate the MCAO-induced ischemic damage and delayed the onset of stroke in aged rats, accompanied by the loss of efficacy in ABR and anti-inflammatory action. On the contrary, Ket enhanced ABR function and delayed the onset of stroke in aged rats. That evidence is parallel to our overall hypothesis, suggesting that intact ABR is required for the neuroprotection of estrogen, and an age-related decline in estrogen regulation on ABR and its downstream α7nAChR anti-inflammatory pathway may be the reasons for estrogen effectiveness in a gradual loss in post-menopausal women, especially those with risk of stroke. The proposed mechanism of estrogen’s age-dimorphic effect on brain ischemic injury is exhibited in Figure 7.

Due to the occurrence of stroke, primarily in the elderly, it is crucial to investigate the function of estrogen in older animals to anticipate its neuroprotectant function (Sohrabji et al., 2013b). In older female animal stroke models, the effect of estrogen treatment is debatable, as some studies revealed a favorable effect (Selvaraj et al., 2018; Burguete et al., 2019; Park et al., 2020; Oppong-Gyebi et al., 2022) and others a neurotoxic or negative effect (Rapp et al., 2003; Selvamani and Sohrabji, 2010; Leon et al., 2012; Manson et al., 2013; Weill et al., 2016; Cespedes Rubio et al., 2018; Tang et al., 2020). Herein, the failure of neuroprotection of estrogen was supported by acute cerebral ischemic damage and the onset of stroke in aged rats, without proinflammatory and neurotoxic effects observed. These differences in animal species, the MCAO model, and the determination of estrus acyclicity may contribute to the discrepant results. Animal species determine susceptibility to stroke and its risk factors, indicating genetic variation. Stroke models have more than one method, which determines the nature of the ischemic insult itself; for example, the MCAO model causes rapid ischemia and reperfusion, while the opposite is true with the vasoconstrictive stroke model conferring gradual ischemia and reperfusion. These differences may crucially result in the variation in the pathophysiology of stroke between studies and predict the estrogen-protective or –non-protective action. In addition, the dichotomized outcome in adult OVX female animals was also reported as the influence of estrogen on stroke (Strom et al., 2011; Leon et al., 2012; Cai et al., 2014; Wang et al., 2014; Zheng et al., 2015). According to a recent systematic review and meta-analysis (Strom and Ingberg, 2014), subcutaneous administration (baseline 17β-estradiol dose of 10–110 pg./ml) had the same neuroprotective effects in the tested dosages, which was in line with our study, i.e., the same range of estrogen dosages, confirming the effects of neuroprotection exhibited in adult OVX female rats.

Aging is a complex process that leads to a modified innate brain environment (Sohrabji et al., 2013a; Honarpisheh et al., 2022), contributing to age-related disorder development, including stroke. The opinion that age-related changes may be synergistic to modify the effects of estrogen treatment is universally accepted. To date, some age-related decline in endocrine cytokines (such as IGF-1 and IGF-1 regulatory component) has been reported to alter the estrogen-therapy effect in older animals and women, as well as the gut microbiome (Selvamani and Sohrabji, 2010; Sohrabji et al., 2013b; Park et al., 2020). However, the current study can not explain all of age-related differences. In the present study, further investigations were made to identify the factors that affect the estrogen neuroprotective effect in the elderly, and the matter was taken from the known stroke risk factors, including hypertension and dysfunction of ABR.

In view of two expected features indicated by accumulating evidence, BRS serves as an ideal candidate. First, BRS exhibited a negative correlation with age and naturally declined in older age, which may be associated with worsened vascular compliance in elderly women (Irigoyen and Krieger, 1998). Second, BRS was regulated by estrogen (Pamidimukkala et al., 2003; El-Mas et al., 2012). Deprivation of estrogen causes baroreflex impairment (De Melo et al., 2016), and replacement of estrogen enhances BRS in post-menopausal women and OVX animals (Hunt et al., 2001). In line with earlier studies, the above two features were also observed in the present study. Estrogen exhibited an age-dimorphic effect both on cerebral ischemic damage and ABR. In the action of age and estrogen, a negative correlation was observed between BRS and cerebral ischemic damage. Furthermore, ABR impairment by SAD caused a significantly aggravated ischemic injury, partly attenuating estrogen-protective effects against stroke in adult rats. Collectively, our observations indicate that an intact ABR is required for the effective protection of estrogen in adult rats. As a baroreflex-positive agent, Ket delayed the onset of stroke in aged female SHRSP. Thus, dysfunction of ABR and unresponsiveness to estrogen in aged female rats are responsible to a reasonable extent for the diminished neuroprotective effect of estrogen.

As regards the estrogen–blood pressure association, contradictory results were previously reported (Ashraf and Vongpatanasin, 2006). Menopause is correlated to a significant elevation in the prevalence of hypertension in women, indicating that estrogen has a protective effect on blood pressure. However, based on clinical trials, a small elevation, rather than a reduction, in SBP was detected with the administration of oral estrogen in post-menopausal women, but no detectable impact was observed in DBP (Ashraf and Vongpatanasin, 2006). Our result is consistent with the clinical evidence and could not account for the loss of the neuroprotective effect of estrogen in aged rats. Notably, the aged rats in the present study did not exhibit hypertension, suggesting that aged rats were not susceptible to hypertension; therefore, differences in the pathology model and animal species may explain the dichotomized discrepancy.

Cerebral ischemia causes a typical inflammatory response following stroke. The post-stroke inflammatory response is involved in the pathophysiology of cerebral ischemia and is affected by ABR and estrogen (Engler-Chiurazzi et al., 2017; Cespedes Rubio et al., 2018; Xu et al., 2022a). In the central nervous system, the vagus nerve is one ABR arm, and by being activated, it can inhibit inflammation through a “cholinergic anti-inflammatory pathway” dependent on α7nAChR (Li et al., 2021). Acetylcholine, the principal vagus nerve neurotransmitter, can limit human-macrophage-produced proinflammatory cytokines dependent on the α7nAChR (de Jonge et al., 2005). Previous evidence suggests that estrogen protects the ischemic brain through anti-inflammatory actions, including the suppression of microglial activation and iNOS-mediated immune response (Engler-Chiurazzi et al., 2017; Cespedes Rubio et al., 2018; Xu et al., 2022a); however, the ABR downstream pathway function, which is a cholinergic anti-inflammatory pathway, has not been investigated previously. In our study, estrogen led to α7nAChR overexpression and reduced proinflammatory cytokine levels in the serum, supporting the enhancement of cholinergic anti-inflammatory signaling; moreover, dysfunction of ABR alleviated these estrogen effects, suggesting that the downstream cholinergic anti-inflammatory signaling participates in estrogen-mediated protection against stroke. As expected, estrogen efficiency in enhancing cholinergic anti-inflammatory signaling was attenuated in aged rats, which is in concert with the results involving the ABR; therefore, we further infer that the unresponsiveness of ABR and its upstream cholinergic anti-inflammatory signaling to estrogen may contribute to its diminished neuroprotection.

The present research offered crucial yet novel insights, but it also had some drawbacks. First, estrogen levels were not evaluated in the brain. There are two groups of estrogen effects with regard to the nervous tissue, those groups that are synthesized peripherally and subsequently penetrate the blood–brain barrier and those groups that are *de novo* synthesized within the brain (Lu et al., 2020). Herein, only peripherally serum estrogen levels were measured; yet, they represent only crude concentration estimates in the brain where the actual effects occur. Second, estrogens function through multiple distinct pathways, including the classical nuclear receptors such as ER-α and ER-β and membrane-linked receptors apart from other molecular mechanisms, thus adding to the complexity of the underlying mechanism. In the present study, the estrogen pathway was not analyzed. Third, the present study did not intensively investigate how estrogen regulates BRS. The reflex center of ABR, i.e., the nucleus tractus solitarii (NTS), was not examined. As regards the two efferent nerves, a previous study about the age-related decline in BRS supported the fact that the main change is initiated from vagal rather than from sympathetic components of BRS (Irigoyen and Krieger, 1998). Comparatively, estrogen was demonstrated to modulate the baroreflex function both by vagal and sympathetic nerves (Hunt et al., 2001; Pamidimukkala et al., 2003). Herein, vagal and sympathetic outflows were not determined, and only downstream of the vagal nerve was neural cholinergic anti-inflammatory signaling explored.

In conclusion, this study demonstrated that ABR is involved in the estrogen’s age-dimorphic effect in IS. In adult female rats, estrogen protects against IS by improving ABR and then activating cholinergic anti-inflammatory signaling. However, estrogen therapy showed no neuroprotective effect on aged female rats, dysfunction of ABR, and unresponsiveness to estrogen, all of which contribute to the loss of estrogen efficacy against cerebral ischemia. Herein, we present innovative insights into the estrogen mechanism in IS, suggesting that ABR is a therapeutic stroke prevention target in post-menopausal women.

## Data availability statement

The original contributions presented in the study are included in the article/supplementary material, further inquiries can be directed to the corresponding author.

## Ethics statement

The animal study was reviewed and approved by the general hospital of Jinan Military Command Ethics Committee.

## Author contributions

LW, JW, QS, HS, and J-MG: participated in the design of the study, performed the experiments, analyzed the data, and drafted the manuscript. J-MG: designed and guided the work. QS: contributed reagents/materials/analysis tools. All authors contributed to the article and approved the submitted version.

## Funding

This study was supported by National Natural Science Foundation of China (81402927 and 81973327).

## Conflict of interest

The authors declare that the research was conducted in the absence of any commercial or financial relationships that could be construed as a potential conflict of interest.

## Publisher’s note

All claims expressed in this article are solely those of the authors and do not necessarily represent those of their affiliated organizations, or those of the publisher, the editors and the reviewers. Any product that may be evaluated in this article, or claim that may be made by its manufacturer, is not guaranteed or endorsed by the publisher.
